# Peer Led Team Learning in Introductory Biology: Effects on Peer Leader Critical Thinking Skills

**DOI:** 10.1371/journal.pone.0115084

**Published:** 2015-01-28

**Authors:** Julia J. Snyder, Jason R. Wiles

**Affiliations:** 1 Department of Biology, Syracuse University, Syracuse, New York, United States of America; 2 Department of Science Teaching, Syracuse University, Syracuse, New York, United States of America; NYIT College of Osteopathic Medicine, UNITED STATES

## Abstract

This study evaluated hypothesized effects of the Peer-Led Team Learning (PLTL) instructional model on undergraduate peer leaders’ critical thinking skills. This investigation also explored peer leaders’ perceptions of their critical thinking skills. A quasi-experimental pre-test/post-test with control group design was used to determine critical thinking gains in PLTL/non-PLTL groups. Critical thinking was assessed using the California Critical Thinking Skills Test (CCTST) among participants who had previously completed and been successful in a mixed-majors introductory biology course at a large, private research university in the American Northeast. Qualitative data from open-ended questionnaires confirmed that factors thought to improve critical thinking skills such as interaction with peers, problem solving, and discussion were perceived by participants to have an impact on critical thinking gains. However, no significant quantitative differences in peer leaders’ critical thinking skills were found between pre- and post-experience CCTST measurements or between experimental and control groups.

## Introduction

Over the past few decades, leading science and science education organizations [1–3] have emphasized the importance of group activity to improve students’ conceptual understanding and more recently, productive engagement in science [4]. This has led to a call for a paradigm shift from traditional, instructor-centered classrooms to student-centered classrooms; providing students an opportunity to be actively engaged in their learning. As emphasized by the Science College Board Standards for College Success [5], group activities for students studying science are of particular importance in providing an opportunity for engagement in science. Nevertheless, at most universities, undergraduate students continue to be enrolled in large lectures in which students are given few opportunities to engage in small group activities.

Peer-Led Team Learning (PLTL) is a pedagogical approach to providing small group instruction that can be employed in conjunction with the traditional lecture component of undergraduate courses that has become so deeply entrenched in university systems. The PLTL approach was developed in the early 1990’s [6] and implemented in undergraduate chemistry courses to allow students to work in problem-solving teams [7]. Since its conception, PLTL has been introduced into other undergraduate science courses, including organic chemistry, biology, and anatomy and physiology [8–10]. In the PLTL model, students work in small groups of six to eight students led by an undergraduate peer who has previously taken and been successful in the course. Peer leaders work collaboratively with the course instructor to facilitate small group problem-solving after being trained in learning theory, pedagogical methods, and the conceptual content of the course [11]. Leaders are not experts in the content, nor are they expected to provide answers to the students in their workshop groups. Rather, they guide and mentor students to develop their own understanding of concepts.

Since the introduction of PLTL, many studies have documented the effectiveness of the PLTL model. Research has focused primarily on the academic benefits to the students who have participated in the PLTL workshops. Studies have shown improvement in students’ performance, attitudes, retention in the course [9, 10, 12–15] conceptual reasoning [16], and critical thinking skills [17], yet little attention has been given to the academic benefits for the peer leaders.

Of the studies that have examined the effects of PLTL on peer leaders of the workshops, few have focused on the benefits reported by leaders during their leadership experience [18, 19]. Through journal entries and survey responses, peer leaders reported benefits such as increased content knowledge, improved communication skills, and increased interest in teaching. In contrast, other studies documented evidence of peer leaders’ benefits based on surveys, focus groups, and individual interviews conducted years after college and the leadership experience had been completed [20–22]. The results of these studies confirmed similar benefits such as reinforcement of the leaders’ prior learning, greater confidence, and improved team-related skills. Peer leader participants in a study by Blake [23] reported perceived gains in their content knowledge. Such gains were confirmed via quantitative analysis of a content quiz given pre- and post-leadership experience which revealed a significant improvement in content knowledge.

Of the few studies that reported on the benefits to peer leaders of the PLTL workshops, most relied only on anecdotal evidence, student self-report [18–21], or superficial analysis of content gains [23]. Whether or not the PLTL training and leadership experience can improve critical thinking in the leaders, as research has shown for the students participating in the workshops [17], has yet to be explored. The goal of this research is to contribute to the body of literature on the potential benefits of PLTL to the peer leaders by exploring what influence the training and leadership experience may have on peer leaders’ critical thinking skills in the context of an introductory biology course at a large, private research university in the Northeastern United States.

### Theoretical Framework

#### Effects of PLTL on Peer Leaders

Like many cooperative learning strategies, the PLTL model encourages students to actively engage in their own learning. Differing from traditional cooperative learning strategies, however, this model provides some guidance to the students in a setting outside of lecture and without teacher intervention [24]. The PLTL model involves teams of six to eight students, often referred to as the workshop, each with a peer leader who has recently and successfully completed the same course in which the students are enrolled. Prior success in the course, as a qualification for peer leaders, is generally defined as having earned a grade of A or B in the course. In addition, peer leaders should demonstrate good communication skills and leadership potential. The peer leaders facilitate group work amongst the team of students and are not responsible for providing answers to any of the problems. Because they are usually neither content experts nor experienced facilitators, peer leaders take part in a leader training program in which the course instructor and an education specialist prepare the leaders to guide student-student interactions [25].

Since the introduction of the model into introductory chemistry courses, the PLTL approach has been implemented into many undergraduate science courses, and its effectiveness has been documented in regards to both students participating in the workshops and peer leaders facilitating the workshops. However, as more attention has been given to the benefits of PLTL for students, the focus of this study is on the comparatively under-researched potential effects of PLTL on peer leaders.

Of the research that has been done regarding the effects of PLTL on peer leaders, most studies have focused on self-report or anecdotal evidence of leader benefits with little information on grade performance or other important collegiate skills, such as improved conceptual reasoning, content knowledge, or critical thinking.

Several studies have focused on the benefits reported by leaders during the leadership experience. Through qualitative data, including journals and surveys, Tenney and Houck [19] revealed five major benefits as reported by the peer leaders: increased content knowledge, improved relationship with course instructor, enhanced teaching skills, improved people skills, and financial compensation. Micari, Streitwieser, and Light [22] confirmed through interviews, surveys, and focus groups that peer leaders thought their cognitive skills improved, as well as their communication and pedagogical skills, while Johnson and Loui [18] found additional benefits of the leaders, including increased self-confidence, increased awareness of intellectual diversity, and increased interest in teaching. While these studies demonstrate clear benefits to the student leaders, long-term benefits such as continued interest in teaching, improved cognitive skills, and continued confidence cannot be confidently claimed.

In contrast to the studies previously discussed, Gafney and Varma-Nelson [20] investigated how graduates who were once peer leaders in the PLTL program viewed their leadership experience several years after college. Results of a Likert-type survey showed that respondents unanimously agreed that their leadership experience was among their most productive learning experiences. Peer leaders felt that not only was their content reinforced but that they had gained confidence in interacting with other people.

Gafney and Varma-Nelson [21] continued their research on the long-term effects of the PLTL experience on peer leaders and found that results confirmed previous evidence that leaders reap benefits such as improving their own learning, developing confidence and perseverance, and improving team-related skills. The findings suggest that leadership experiences, such as the PLTL experience, lead to personal and professional benefits even after college is complete.

Unlike the other studies on peer leadership which only reported perceived student benefits of the PLTL training and leadership experience, Blake [23] quantitatively measured some of the leader-reported content knowledge gains. Student leaders from the general chemistry course at a Midwestern university participated in the study. Results of a pre- and post-test indicated that student leaders did increase their knowledge of chemistry content. Based on these encouraging, if somewhat superficial, findings, the author suggests that being a workshop leader provides critical reinforcement of key concepts that prevents the typical deterioration of knowledge over time.

#### Critical Thinking

Within the literature on critical thinking, a uniformly recognized and universally accepted definition of the construct does not exist. Critical thinking overlaps with many constructs including reflective thinking, creative thinking, problem-solving, higher-order thinking, and metacognition. The lack of consensus on a collective definition of critical thinking comes from the combination of philosophical and psychological theories underlying the construct, yet various definitions share common aspects. Without a clear conceptualization of critical thinking, it has been difficult for researchers and practitioners to establish appropriate instructional interventions and instruments to appropriately assess critical thinking. Research on the most effective instructional strategies for enhancing students’ critical thinking has been inconclusive [26].

By combining many philosophical and psychological theorists’ definitions, the American Philosophical Association (APA) Delphi Panel yielded the following conceptualization of critical thinking that characterizes a set of cognitive skills and the disposition toward using them:

We understand critical thinking to be purposeful, self-regulatory judgment which results in interpretation, analysis, evaluation, inference, as well as explanation of the evidential, conceptual, methodological, criteriological, or contextual considerations upon which that judgment is based…The ideal critical thinker is habitually inquisitive, well-informed, trustful of reason, open-minded, flexible, fair-minded in evaluation, honest in facing personal biases, prudent in making judgments, willing to reconsider…and persistent in seeking results which are as precise as the subject and the circumstances of inquiry permit…[27].

This conceptualization of critical thinking provided a framework for instructional interventions and appropriate assessments of critical thinking skills which could inform teachers about their success in teaching students to think critically [28] and led to the development of the standardized California Critical Thinking Skills Test (CCTST) to measure six cognitive skills identified by the Delphi panel as essential [29].

Because the APA definition of critical thinking is based on the most current consensus definition of critical thinking that includes cognitive skills and dispositions and was used as the theoretical basis for designing the CCTST, this was the definition adopted for this study; to determine if the PLTL model can enhance critical thinking skills of undergraduate peer leaders.

Since the 1980’s, a primary goal of colleges and universities has been to help students develop the ability to think critically [26, 30]. Critical thinking is a skill that will last throughout a lifetime, regardless of what content may or may not be forgotten. Being an independent, critical thinker will allow individuals to be contributing citizens to their profession and society, long after their academic career is over. Fostering critical thinking skills requires colleges and universities to adopt effective instructional strategies that will enhance such skills. For this reason, much research has been devoted to determining what teaching approaches inhibit or enhance students’ ability to think well.

While there is a vast amount of research on the teaching of critical thinking skills, either explicitly or through a conducive classroom environment, instructional interventions that can enhance critical thinking are generally consistent. Regardless of the approach to teaching critical thinking, the literature reveals several classroom activities that have been shown to enhance critical thinking, including faculty and peer interaction [31–36]), writing tasks and class discussions [33, 37], and problem-based learning activities [38]. Additional research found that science courses, active learning techniques, and the number of hours spent per week studying had a positive impact on critical thinking [39, 40].

These studies are important to consider with regard to the PLTL model as they indicate that students’ participation and interaction with peers can positively influence critical thinking skills. Peer leaders are involved not only in participation of the class they are facilitating, but they are also involved in direct participation with their peers and instructor in the weekly meetings in which they themselves have to work through various problems they will assign their own group of students. In addition to adding a science course to their program of studies, leaders would be required to spend more hours studying the material by not only spending extra hours in the workshop training but also in preparation for the workshop itself.

Within the PLTL leadership training meetings and the workshops themselves, peer leaders are actively involved in discussion with other students. They have the opportunity to receive feedback from peers providing them an environment to foster critical thinking. In addition, leaders may improve critical thinking skills through the writing process of keeping their weekly journals.

Abrami et al. [41] performed a recent meta-analysis of the empirical evidence on the impact of instruction on students’ critical thinking skills. The mixed method, in which critical thinking is taught independently within a specific content course, instructor training, and collaborative learning conditions were among specific interventions found to have a positive effect on development of students’ critical thinking skills. Based on these findings, the authors suggest that educators take steps to make critical thinking objectives explicit in courses and include them in training programs for faculty.

In another review of methods and conceptions of teaching that are likely to inhibit or enhance critical thinking, Pithers and Soden [42] included simply agreeing or disagreeing, merely demonstrating and explaining, cutting off student responses, using basic recall questions, and teacher beliefs that the “right” answer is important as inhibitory teaching approaches. In contrast, methods thought to enhance critical thinking included teaching from multiple perspectives, focusing on linkages in content to explore various themes, and reflection and analysis of students’ core ideas. Pithers and Soden also discussed metacognitive approaches to help students to think well. Modeling ways of thinking and scaffolding were among the approaches to help students to learn to think as they learned their discipline. Scaffolding is a process in which the teacher moves the student’s thinking forward through a series of systematic questions that lead the student to understanding.

Developing critical thinking can be accomplished more successfully with a student-centered approach to teaching and learning. The review above specifically mentions small-group tutorials and well-designed problem based courses as likely avenues for encouraging critical thinking. In the PLTL instructional model, peer leaders are involved in a small-group, student-centered instructional model in which they are active participants in a weekly meeting where a scaffolding process helps them in the process of solving content-related problems.

One such study on problem-based learning (PBL) and critical thinking has been conducted and found to be an educational strategy that promotes critical thinking. Tiwari, Lai, So, and Yuen [38] compared the effects of PBL and lecturing approaches on the development of students’ critical thinking. Based on the California Critical Thinking Disposition Inventory results, PBL students had significantly higher overall critical thinking disposition (motivation to value and utilize critical thinking) scores than the lecture students on completion of the course. Higher scores continued for two years afterward. While self-report by students and limiting the study to nursing students makes it impossible to generalize, the findings are consistent in suggesting that PBL can improve critical thinking.

Studies of the effectiveness of PBL are of particular importance to PLTL because they are similar pedagogies. Although PBL was developed and utilized primarily in medical education within the construct of the lecture, adaptations were made to implement the instructional model into the college and university setting. With its basis in constructivism and the social aspects of learning as indicated by Vygotsky [43], PLTL, just as PBL, situates students in their zone of proximal development by presenting challenging problems that they can’t solve easily on their own but can by interacting with peer members of the team [44]. If the student-centered pedagogical approach of PBL can be effective at improving critical thinking skills, placing students in a PLTL setting may also improve critical thinking. PLTL allows students to work with a peer leader who is closer to their own ZPD than a course instructor and provides the opportunity for students to work with a peer team on challenging problems that they cannot easily solve on their own. The peer leader provides more course structure and content knowledge than current students and is capable of facilitating participation and peer interaction among the other students.

### PLTL and Critical Thinking

Of the many studies that have examined the effectiveness of the PLTL model, only one tested the model as a predictor of critical thinking gains. Quitadamo, Brahler, and Crouch [17] examined the impact of PLTL on critical thinking gains in six undergraduate science and math courses at a research university in the Pacific Northwest. Results showed that the PLTL model had a positive impact on critical thinking gains in science but not in math courses, regardless of gender, ethnicity, or other variables. In addition, grade performance and retention improved, particularly for females. Based on these findings, the authors suggest that continued development of the PLTL model may serve to increase critical thinking gains in undergraduate students. Research on the leadership experience of PLTL on critical thinking gains has yet to be explored.

As evident from the previous literature discussion on PLTL and critical thinking, peer leaders are engaged in many activities that have been shown to improve critical thinking skills. Peer and faculty interaction, class discussion, and writing tasks are involved in their collaborative leadership experience. Instructors utilize a scaffolding approach to model how leaders should help their own students with their thought processes, and leaders are engaged in weekly problem solving sessions in which they construct their own understanding. Therefore, we hypothesized that the PLTL training and leadership experience should lead to better critical thinking skills.

In order to test this hypothesis, we conducted an experiment in the context of a mixed-majors, introductory-level biology course at a large university guided by the following research questions:

1. Does the PLTL training and leadership experience in biology influence the critical thinking skills of undergraduate peer leaders?
Sub-Question: Does the PLTL training and leadership experience in biology influence any particular critical thinking skill tested by the CCTST (analysis and interpretation, evaluation and explanation, inference, deductive reasoning or inductive reasoning) more than another?
Sub-Question: Does peer leader perception of critical thinking correspond to actual CCTST results?
2. What differences in critical thinking skills, if any, exist, between student leaders in the PLTL instructional program and similar students without the PLTL training and leadership experience?
Sub-Question: How does peer leader perception of critical thinking compare to control group participants’ perception of critical thinking?
3. Does the PLTL training and leadership experience in biology influence critical thinking skills of undergraduate peer leaders, controlling for the effects of demographic variables?
Sub-Question: Which variables have the greatest impact on critical thinking gains?

## Methods

### Design

A quasi-experimental pre-test/post-test with control group design was employed to determine critical thinking gains in PLTL leaders and a control group comprising similar students who were unable for various reasons, but otherwise eligible, to serve as PLTL leaders. The PLTL training and leadership experience was the independent variable identifying the experimental group to determine if the dependent variable, critical thinking, improved as a result. To minimize validity threats of history, maturation, regression, and selection, a critical thinking post-test was given approximately 15 weeks after the pre-test to both the experimental and control groups.

### Ethics Statement

This research study was approved by the Syracuse University Institutional Review Board. Prior to conducting the research, participants were provided with a written consent form explaining the details of the study, including both benefits and risks to participating. All participants indicated their consent to participate in the study by signing the consent form and returning it to a non-instructor/researcher third party. For privacy protection, each voluntary participant was assigned a unique 9-digit identification number by the third party administrator to use on all data collection instruments.

### Participants

In keeping with the conventional PLTL model, undergraduate students who had obtained a final course grade of A or B in the second semester of introductory biology at a large, research university in the Northeastern United States were invited to participate in the study. Of the 75 interested participants, two groups were established: one group of students who were interested and able to complete the requirements of being a PLTL leader (experimental group) and one group of students who were interested but not able to be a leader due to various time/schedule constraints (control group).

Interested participants who were 18 years of age or older completed a demographic survey to determine information regarding control variables for the data analyses of the study. Tables 1, 2, 3, and 4 provide the demographic make-up of the participants. While gender distribution of the two groups of participants was evenly matched, the PLTL group was comprised primarily of juniors while the non-PLTL group was comprised primarily of sophomores. About 60% of the participants were white/Caucasian, and approximately 40% were non-white including Hispanic/Latino, African American, Asian, and other racial groups. Racial/ethnic categories were those used by the university in this study. 80% of the participants were science majors. Over 60% of them had taken more than 6 science courses prior to the start of the academic term, and almost 75% of the participants took 2, 3, or 4 science courses during the academic term.

**Table 1:** Demographics for PLTL and Non-PLTL Groups (Class standing & Gender).

**Method**	**n**	**Class Standing (%)**				**Gender (%)**	
		**Fr**	**So**	**Jr**	**Sr**	**M**	**F**
**PLTL**	37	0	38	51	11	43	57
**Non-PLTL**	17	0	75	19	6	41	59
**Total**	54	0	56.5	35	8.5	42	58

**Table 2:** Demographics profile for PLTL and Non-PLTL Groups (Ethnicity).

**Method**	**n**	**Ethnicity (%)**				
		**White**	**Black**	**Hispanic/Latino**	**Asian**	**Other**
**PLTL**	37	76	5	8	8	3
**Non-PLTL**	17	47	18	18	12	6
**Total**	54	61.5	11.5	13	10	4.5

Racial categories used by the university in this study.

**Table 3:** Major and Number of Science Courses taken Prior to Spring 2011 for PLTL and Non-PLTL Groups.

**Method**	**n**	**Major (%)**		**Number of Science Courses Prior to Spring 2011 Term (%)**					
		**Science**	**Non-Science**	**2**	**3**	**4**	**5**	**6**	**6+**
**PLTL**	37	84	16	3	5	5	0	14	73
**Non-PLTL**	17	76	24	18	0	12	18	6	47
**Total**	54	80	20	10.5	2.5	8.5	9	10	60

**Table 4:** Number of Science Courses taken During Spring 2011 for PLTL and Non-PLTL Groups.

**Method**	**n**	**Number of Science Courses During the Spring 2011 Term (%)**							
		**0**	**1**	**2**	**3**	**4**	**5**	**6**	**6+**
**PLTL**	37	0	19	32	27	16	0	3	3
**Non-PLTL**	17	6	12	24	24	24	0	6	6
**Total**	54	3	15.5	28	25.5	20	0	4.5	4.5

### Procedure

During the first week of classes, the valid and reliable California Critical Thinking Skills Test (CCTST) was administered to all participants in the experimental and control groups. At the end of the semester, the same test was administered as a posttest, along with an open-ended questionnaire targeting participants’ perceptions of critical thinking and how it changed throughout the semester.

### Instructional Intervention

The instructional intervention was composed of (a) peer leader training and (b) peer-led workshops throughout the semester. The potential impact of the PLTL training and leadership experience on critical thinking skills was dependent on leaders’ workshop practices.

#### Leader Training

A two-credit course was offered that related educational research literature on students and learning to classroom applications in problem-solving activities. The peer leaders met with a learning specialist for one 55-minute class each week for 13 weeks to discuss teaching and learning theory and how to apply it conceptually, debrief on previous weeks’ sessions, and practice problem-solving strategies by collaboratively working on instructor-generated content problems [25].

During the first class, the learning specialist discussed the PLTL instructional model. Peer leaders were also provided with a PLTL leader handbook [45] that included many assigned readings on learning theory and group dynamics.

At the start of each class succeeding the first, the learning specialist would either do a short activity related to the weekly reading assignment or ask if there were any questions about the assigned readings. Peer leaders were then able to debrief on the previous weeks’ sessions. During the debriefing time, peer leaders would share personal experiences with the other leaders, as well as the learning specialist. They would both offer and receive suggestions on how to handle various issues that arose during their PLTL workshops.

Following the debriefing time, the learning specialist passed out a problem set for the week. Problem sets were developed based on the weekly content covered by the Biology instructor. Each problem set had several activities for the workshop students to engage in. The learning specialist modeled the problem-solving methods each week by having the peer leaders collaborate in small groups on the problem-solving activities much like their students should. While answers were generally not provided to the problem set activities, peer leaders were able to ask for clarification or helpful hints on any of the activities.

#### Peer-Led Workshops

After the leader training class each week, the peer leaders facilitated a one-hour PLTL session for a group of students of their own (between one and ten, depending on how many students attended the workshop), without the presence of the instructor or learning specialist. Leaders offered guidance and support to their own students through the thought processes of solving the same problems they themselves worked on in their class with the learning specialist and other leaders.

Peer leaders were required to keep a weekly written journal of their PLTL workshop experiences. Journal entries were submitted within three days of the workshop time. While journal entries varied among the leaders, peer leaders generally reflected on how their workshop went for the week. Reflections included how they thought the workshop went, what problems the students had, how the students interacted with each other and with them, etc.

In addition to the journal entries, peer leaders were required to work with a partner and develop a problem set based on the teaching and learning strategies discussed throughout the PLTL leader training course. The problem set had to be a topic that had not been covered in a previous problem set or that their students had had difficulty with previously in the semester.

### Data Analyses

Statistical analyses of the quantitative data (S1 Dataset) associated with the CCTST were performed through a statistical software package, SPSS 19. Among the experimental group participants, a one-way repeated measures analysis of variance (RM ANOVA) was conducted to analyze overall critical thinking scores. Paired t-tests were also performed for each of the five sub-scale scores to examine differences in critical thinking skills as measured by the CCTST prior to the instructional intervention and immediately following the PLTL training and leadership experience. To increase statistical accuracy, gender, ethnicity, class standing, major and number of science courses taken prior to and during the Spring 2011 semester were analyzed as co-variables.

Overall critical thinking scores were compared between the PLTL group and the non-PLTL group using mean, standard deviation, and two-way RM ANOVA. The two-way RM ANOVA was applied to the data because there were paired pre- and post-test scores, as well as a comparison group. Although the total pre- and post-test scores of the PLTL and control groups were normally distributed, there was a non-normal distribution of data in either the pre-test or the post-test of the five sub-scales of critical thinking. For this reason, nonparametric analyses of the five sub-scales were conducted.

Qualitative data obtained through the open-ended critical thinking questionnaire was reviewed and analyzed by coding the responses of the participants as “perceived improvement in critical thinking skills” or “did not perceive improvement in critical thinking skills” to determine if participant self-report coincided with actual critical thinking test score data. Additional codes of participants’ responses were developed on the basis of emerging information and further analyzed to identify common themes about factors perceived to influence critical thinking skills.

## Results

Herein, findings of the influence of the PLTL training and leadership experience on undergraduate peer leaders are presented for both overall critical thinking skills scores, as well as five sub-scale scores. Results of a comparison of peer leader perception and actual critical thinking skills are also presented, as well as findings of analyses controlling for demographic factors.

### Influence of PLTL on Critical Thinking Skills

Prior to the PLTL training and leadership experience, participants’ critical thinking skills were measured with the CCTST. The output from the CCTST was provided as a score between 0 and 34, with a higher score indicating a higher level of critical thinking. Individual scores for each of the five sub-scales were also provided. The mean pre-test score on the CCTST was 18.46 (n = 37). The mean on the CCTST post-test was 18.76 (n = 37), indicating an average gain of 0.30 over CCTST pre-test scores. A one-way repeated measures ANOVA was conducted to compare CCTST pre-test and post-test scores, and no significant difference was observed for critical thinking skills of undergraduate peer leaders participating in the PLTL training and leadership experience, F(1, 36) = 0.290, p = 0.593, power = 0.082, partial eta squared = 0.008.

Paired t-tests were conducted to compare the pre-test and post-test scores of the five critical thinking sub-scales, and no significant differences were revealed for any of the five critical thinking sub-scales: inductive reasoning, *t* (36) = .356, p = .724, deductive reasoning, *t* (36) = −1.061, p = .296, analysis and interpretation, *t* (36) = .780, p = .440, inference, *t* (36) = −.643, p = .525, and evaluation and explanation, *t* (36) = −.919, p = .364. Tables 5 and 6 present the means (with standard deviations in parentheses) of the sub-scale scores.

**Table 5:** Influence of Method on Critical Thinking Sub-Scale Scores (Inductive Reasoning, Deductive Reasoning, Analysis & Interpretation).

**Method**	**n**	**Critical Thinking Sub-Scale Scores**					
		**Inductive Reasoning (pre)**	**Inductive Reasoning (post)**	**Deductive Reasoning (pre)**	**Deductive Reasoning (post)**	**Analysis & Interpretation (pre)**	**Analysis & Interpretation (post)**
**PLTL**	37	10.03(1.99)	9.89(2.07)	8.43(2.35)	8.86(2.89)	4.05(1.15)	3.84(1.34)
**Non-PLTL**	17	8.29(1.76)	8.82(3.58)	9.53(3.00)	7.53(3.11)	4.41(1.50)	3.65(1.46)
**Total**	54	9.48(2.07)	9.56(2.65)	8.78(2.60)	8.44(3.00)	4.17(1.27)	3.78(1.37)

Standard deviations are in parentheses.

**Table 6:** Influence of Method on Critical Thinking Sub-Scale Scores (Inference, Evaluation & Explanation).

**Method**	**n**	**Critical Thinking Sub-Scale Scores**			
		**Inference (pre)**	**Inference (post)**	**Evaluation & Explanation (pre)**	**Evaluation & Explanation (post)**
**PLTL**	37	9.62(2.30)	9.86(2.15)	4.78(1.38)	5.05(1.81)
**Non-PLTL**	17	9.71(2.20)	8.71(3.08)	3.71(1.83)	4.00(2.42)
**Total**	54	9.65(2.25)	9.50(2.51)	4.44(1.60)	4.72(2.06)

Standard deviations are in parentheses.

With respect to peer leader perception of critical thinking, 62% of the peer leader participants thought they improved their critical thinking skills over the course of the Spring 2011 semester, while only 43% of the peer leaders actually improved their skills from the pre-test to the post-test. Further analysis of the responses provided on the open-ended questionnaire revealed recurring themes related to participants’ perception of their critical thinking skills. When asked the questions “What do you think contributed to your change in critical thinking skills?” and “How did your experience as a workshop leader influence your critical thinking skills?”, three common themes emerged from the participants’ responses: interacting with others, utilizing different approaches to learning, and problem solving.

First, the participants’ perceived that interacting with peers during the workshops influenced their critical thinking skills. Although the number of students in each workshop varied from week to week, no students were alone to work on the problem sets. Some of the participants shared these responses: “Working with and helping random people through something I’ve succeeded in”; “Working through problem sets with students and observing their way of doing things”; “The peer leader session where you help others get answers while you think about what’s the best way for them to reach the answer without you telling them”; and “Listening to students’ answers and their reasoning behind each answer definitely influenced my critical thinking skills”.

The second theme that emerged was that critical thinking skills improved because of utilizing different approaches to learning. While one participant thought that the PLTL sessions contributed to improvement in critical thinking skills because “it allowed me to apply different types of thinking each week”, two other participants believed improvement was the result of PLTL giving new and different ways to approach biology questions. Even some participants’ who did not perceive an improvement in their critical thinking skills responded that the PLTL sessions taught them another way to approach questions and encouraged them to consider alternative ways of teaching and learning the material.

The third theme that emerged from the questionnaire responses was the emphasis on problem solving as a means of improving critical thinking skills. For example, one participant responded “watching my students work thoroughly through their problem sets and discuss answers with one another made me re-think how I answer questions. Working through PLTL I was able to improve my problem solving skills”. Other participants concurred that leading and working through the problem sets with students contributed positively to their critical thinking skills.

In addition to responses framed in terms of working with others, utilizing various approaches, and working through problem sets, one participant responded that having to elicit discussion in the PLTL group contributed to improved critical thinking skills.

Other questions on the open-ended questionnaire asked peer leader participants, “how do you think you did on the critical thinking pre-test compared to the critical thinking post-test?” and “how have your critical thinking skills changed since the beginning of the semester?” Several students responded that they did better on the post-test because they had seen the questions before and had time to think about them or that they remembered the questions from the pre-test. For the same reason, other students believed that they did worse on the post-test because they simply tried to remember what they wrote the first time instead of thinking the questions through. Being tired, having too many tests this time of year, and not reading as carefully were other reasons provided by participants for not doing as well on the post-test.

In general, peer leader responses to these two questions revealed that students who reported they did worse or the same on the post-test also reported that their critical thinking skills did not change. Similarly, student responses reporting that they did better on the post-test were typically followed by responses suggesting their critical thinking skills had improved. Three peer leaders reported that they did not improve on the post-test but improved their critical thinking skills, while two peer leaders reported that they did better on the post-test but did not improve their critical thinking skills.

A series of one-way repeated measures of ANOVA revealed that class standing, gender, ethnicity, major, number of science courses taken prior to the academic term, and number of science courses taken during the academic term were not indicators of critical thinking gains of peer leaders.

When comparing peer leaders to similar students that did not participate in the PLTL training and leadership experience, the mean pre-test score of the peer leaders on the CCTST was 18.46 (n = 37) and the mean pre-test score of similar students without the PLTL training and leadership experience on the CCTST was 17.82 (n = 17). The mean post-test score of the peer leaders was 18.76 (n = 37), indicating an average gain of 0.30 over CCTST pre-test scores. The mean post-test score of similar students without the PLTL training and leadership experience was 16.35 (n = 17), indicating an average negative gain of 1.47. A two-way repeated measures ANOVA was conducted to compare total pre-test and post-test scores of the experimental and control groups, and no significant interaction was observed for critical thinking skills of peer leaders participating in the PLTL training and leadership experience, F (1, 52) = 2.440, p = 0.124, power = 0.335, partial eta squared = 0.045.

Friedman’s chi-square test revealed no significant differences across groups for deductive reasoning, x^2^ (1) = 0.556, p = 0.456; inductive reasoning, x^2^(1) = 0.023, p = 0.879; analysis and interpretation, x^2^ (1) = 2.632, p = 0.105; inference, x^2^ (1) = 0.220, p = 0.639; and evaluation and explanation, x^2^ (1) = 0.818, p = 0.366 (Tables 5 and 6).

With a small starting sample of non-PLTL leaders, only two participants completed the questionnaire related to perception. Both non-PLTL leaders perceived that their critical thinking skills did not really improve, however, both participants’ critical thinking skills showed a slight improvement from the pre-test to the post-test. Although 62% of the peer leader participants thought they showed an improvement in critical thinking skills while only 43% actually did, with only two participants in the non-PLTL leader group, accurate comparisons could not be made between groups.

## Discussion

The purpose of this study was to investigate whether the PLTL training and leadership experience in Introductory Biology could promote critical thinking in undergraduate peer leaders. The PLTL training and leadership experience provided a collaborative instructional opportunity for peer leaders to interact with faculty and peers while applying learning theory and working through problems related to the content of an introductory biology course. Quantitative analyses demonstrated the impact of the PLTL training and leadership experience on the critical thinking skills of peer leaders, while qualitative data provided insight into peer leader perception of critical thinking skills.

Results of the total pre and post measurements of the CCTST indicated no statistically significant difference in peer leaders’ critical thinking skills. Additionally, results of the five sub-scales of critical thinking as measured by the CCTST indicated no significant differences in peer leaders’ critical thinking skills.

The peer leaders did not have a positive significant improvement in their critical thinking skills, rather their average from pre- to post-test increased slightly. The mean score of the national comparison group on the CCTST was 16.8 [46], while the mean pre-test score of the peer leaders in this study was 18.46. This illustrates that on average, the peer leaders in this study already had good critical thinking skills. With most of the peer leaders being juniors and many being science majors, many had taken a large number of science courses prior to the PLTL training and leadership experience. As discussed in the literature review informing our theoretical framework, the number of science courses taken by an individual can influence critical thinking skills, and this could be a contributing factor as to why this particular group of peer leaders had an average greater than the national comparison group. However, even with a higher starting mean than the national average of the comparison group, according to the CCTST manual, the peer leaders should have been capable of benefiting from an educational model such as PLTL because their score fell in the midrange of total scores [46].

Although the mean pre-test score fell within the midrange of scores on the CCTST and is indicative of persons suitable for learning development with appropriate instructional guidance, peer leader commitment throughout the PLTL training and leadership experience could have played an important role in critical thinking gains. With 37 peer leaders, there was likely to be a high level of variance in the PLTL leadership and training experience. While some leaders may have been actively engaged in class discussion, writing tasks, and interaction with faculty and peers throughout the Biology 200 course and problem solving sessions, others may not have integrated these factors that could potentially influence their critical thinking skills into their experience as well. Some peer leaders may have taken on the role of a facilitator in the workshops without actually participating themselves. One peer leaders’ response that his participation as a PLTL leader did not influence his critical thinking skills much “because the students did most of the thinking, not me” suggests a lack of active participation with the students in the problem solving session. Other peer leaders may have devoted time to discussing study tips or review of material for exams instead of completing the problem sets each week, or they may have simply answered the questions in the problem sets without implementing the specific instructions provided for each group of problems that incorporated the factors that have been shown to improve critical thinking skills. This could explain why some peer leaders improved their critical thinking skills while others did not.

In addition to the high variance in peer leader commitment to the PLTL model, peer leaders’ dispositions to utilize critical thinking skills could have contributed to the lack of significant improvement in critical thinking skills between the pre- and post-test measurement. As discussed in the previous literature review, disposition refers to the consistent internal motivation to engage problems and make decisions by using critical thinking [47]. While some peer leaders may have improved their critical thinking skills during the PLTL training and leadership experience, that improvement may not have been reflected in their post-experience CCTST score if they were not willing to utilize those skills.

Lack of motivation or willingness to engage in the problems on the post-experience CCTST with sufficient effort may have been the result of when the test was administered. Several students commented on their open-ended questionnaire that there were too many tests at that time of the semester or that they were too tired to take the test when given. Others commented that they simply did not read the questions on the post-test as carefully as they read the questions on the pre-test or that they just tried to remember their previous answers. These reasons suggest that some participants may not have been utilizing their critical thinking skills to the best of their ability and could potentially explain the lack of improvement or even negative critical thinking gains achieved by some of the peer leaders.

Interestingly, although only 43% of the peer leaders improved their critical thinking skills, 62% of the peer leaders perceived that their critical thinking skills improved. Previous research demonstrated that peer leaders perceived benefits from the PLTL training and leadership experience such as increased content knowledge, improved people skills, improved confidence and improved cognitive skills [18–22]. These data confirm previous findings that students perceive benefits from the PLTL experience by demonstrating that peer leaders also perceive improvement in their critical thinking skills. However, the students’ reported perceptions do not appear to correspond to measured results in all cases.

A greater percentage of perceived improvement compared with actual improvement may have been attributed to a lack of understanding of the definition of critical thinking by the peer leaders. While peer leaders perceived improvement in their critical thinking skills, they may have defined those skills in relation to how they did on the CCTST or as improved communication skills, collaborative skills, content knowledge, etc. However, with regard to factors that may influence critical thinking skills as identified through the previous literature review, peer leaders identified at least some of those factors as having influenced their critical thinking skills. While the most commonly cited reason for improved critical thinking skills was working with peers, many others cited working through the problem sets as influential in their change in critical thinking skills from the pre- to the post-experience measure of critical thinking. Discussion was also cited as a contributing factor to improved critical thinking skills.

When peer leaders were compared to similar students who did not participate in the PLTL training and leadership experience, results of the total pre- and post-experience measurements of the CCTST, as well as the five sub-scales of critical thinking, indicated no significant differences between the groups. While a small gain in the mean overall critical thinking skills was shown for the peer leaders, a trend of negative gains in the mean overall critical thinking skills was seen for similar students who did not participate in the PLTL training and leadership experience. Just as the peer leader participants’ average, the average critical thinking score of the non-peer leaders was above the mean for the national comparison group for the pre-test, however, the mean on the post-test score dropped below the national comparison group mean. While there was no attrition in the number of participants in the experimental group, there was greater than a 50% attrition rate in the control group of the study. With such high attrition, the sample size of the control group was very limited. Within the small number of participants in the control group, several also had fairly large negative gains in critical thinking implying that the test may not have been taken with sufficient effort. These reasons suggest why the control group may have seen negative critical thinking gains from pre- to post-measurements.

When comparing peer leader perception to non-peer leader perception of critical thinking skills, 62% of peer leaders perceived improvement in their critical thinking skills compared to 0% of the non-peer leaders. This suggests that participation in the PLTL training and leadership experience can influence how students perceive their critical thinking skills. While it is unclear which aspects of the PLTL training and leadership experience influenced this perception, it is reasonable to suspect that the collaborative nature of working through problem sets with peers supported their perception of improved critical thinking skills. However, only two control group participants completed the open-ended questionnaire, so accurate comparisons between the two groups were not possible.

Demographic factors did not appear to be indicators of critical thinking skills or gains therein. Within the peer leader group, the academic major of the participants was the only demographic variable that even approached significance. No significant differences in critical thinking skills were found between genders, class standing, ethnicity, or number of science courses during and prior to the Spring 2011 academic term.

Although critical thinking skills of peer leaders did not have a significant positive improvement, the results of this study seem to indicate that peer leaders view the PLTL training and leadership experience as a positive influence on their critical thinking skills and that the students within their workshops benefit from the PLTL model. None of the participants in this study, no matter how much or how little they improved their critical thinking skills, perceived the PLTL training and leadership experience to affect them negatively. Even if they thought it did not improve their critical thinking skills, several leaders reported that other skills improved, including new ways of approaching problems, communication, increased knowledge, and increased confidence. This confirms previous findings [18–22] and suggests that the PLTL training and leadership experience is of benefit to the peer leaders and their students, even if it does not significantly improve critical thinking skills. With the implementation of the PLTL model for the first time and with no prior studies done on the influence of PLTL on peer leaders’ critical thinking skills, it is premature to conclude that PLTL cannot promote critical thinking in undergraduate biology peer leaders. Although Preszler [48] was not studying critical thinking, he found that refinements of the workshop activities that occurred between the first and third workshop semesters increased the impact of the workshops on student learning, bringing the learning up beyond the levels seen in pre-workshop semesters. Similarly, refinements in the implementation of the PLTL training and leadership experience could lead to improved critical thinking skills of the peer leaders.

A major implication of this study is the impact of the PLTL approach on the concern of shifting students from the traditional instructor-centered paradigm to a student-centered paradigm [15]. The PLTL training and leadership experience is important in educating peer leaders about how to help students actively construct their own understanding of scientific concepts. As the peer leaders facilitate PLTL workshops, they serve as role models to the students of how to utilize various ways of thinking and problem-solving techniques. This teaches the student what a student-centered classroom should look like. As students and peer leaders go on in their academic careers, they may be presented with teaching and leadership opportunities, and exposure to the student-centered PLTL model provided both peer leaders and their students with an alternative pedagogical approach to the traditional instructor-centered paradigm.

Another implication of this study is the potential impact of the PLTL model on retention of students in the sciences. As discussed in the literature review, many studies found that the PLTL model resulted in improved retention of students in introductory level science courses [10, 13, 15, 49]. Retaining students in these introductory, “gatekeeper” science courses is necessary for retaining students in the sciences. In two programs designed to increase retention of female and underrepresented minorities, Monte, Sleeman, and Hein [50] found a second benefit; the retention of the peer mentors was improved. Attracting talented undergraduate students to become peer leaders in a PLTL instructional model may also lead to improved retention of the peer leaders, in addition to improved retention of their students.

Although the focus of this study was not on retention of the peer leaders, there was an overall peer leader retention rate of 100% throughout the training and leadership experience. As illustrated by the participant demographics, peer leaders included males and females, as well as underrepresented minorities. Longitudinal studies of the long-term effects of the PLTL instructional model on retention of students in the sciences should be conducted.

In conclusion, the PLTL training and leadership experience appears to be an effective means of bringing about change in large, undergraduate science courses. The experience is a financially feasible way of equipping undergraduate peer leaders to help students take ownership of their own learning, while providing working relationships among peer leaders and faculty. Additionally, the experience has the potential to improve long-term retention of peer leaders and their students in the sciences.

## Supporting Information

S1 Dataset Peer Leader and Control Group Demographics and Critical Thinking Scores. (XLSX)
